# Unveiling Renal Lipid Deposition: A Rare Case of Hepatic Glomerulosclerosis Resembling Lecithin-Cholesterol Acyltransferase (LCAT) Deficiency Post Liver Transplantation

**DOI:** 10.7759/cureus.64004

**Published:** 2024-07-07

**Authors:** Aseel Zghayer, Ewa Borys, Maria M Picken, Kavitha Vellanki

**Affiliations:** 1 Department of Nephrology, Loyola University Medical Center, Maywood, USA; 2 Department of Pathology, Loyola University Medical Center, Maywood, USA

**Keywords:** liver transplant, lipid deposits, hepatic glomerulosclerosis, lcat deficiency, liver cirrhosis

## Abstract

Hepatic glomerulosclerosis, a renal complication of liver cirrhosis, presents challenges in diagnosis and management. This case report discusses the rarity of kidney biopsy findings resembling lecithin-cholesterol acyltransferase (LCAT) deficiency post liver transplantation. We present the case of a patient with end-stage liver disease (ESLD) from alcohol-related cirrhosis, who underwent orthotopic liver transplantation (OLT) with persistent proteinuria after transplantation. Kidney biopsy revealed features of hepatic glomerulopathy resembling both IgA nephropathy (IgAN) and LCAT deficiency. The histopathological similarities between hepatic glomerulosclerosis and LCAT deficiency suggest a potential link between liver disease and lipid deposition in the kidneys. The clinical course and outcomes of such renal alterations post liver transplantation remain uncertain, highlighting the need for further research in glomerular pathology in the context of liver transplantation. This case underscores the importance of kidney biopsy in ESLD patients and the necessity for more attention to glomerular pathology post liver transplantation, especially in the current era of increasing liver transplantation rates.

## Introduction

Hepatic glomerulosclerosis, a term introduced by Bloodworth and Sommers in 1959 [1], refers to renal glomerular alterations associated with liver failure and cirrhosis. This phenomenon often presents asymptomatically, making detection reliant on the observation of proteinuria or abnormal urine sediment [2]. These alterations typically involve the gradual thickening of capillary walls and expansion of the mesangium within the renal structure. Electron microscopy (EM) reveals the presence of finely granular deposits beneath the endothelium, within the mesangium, and along the basement membrane. As the condition progresses, there is a noticeable increase in the mesangial matrix and thickening of the basement membrane, ultimately leading to the progressive obliteration of the glomerulus [3].

IgA nephropathy (IgAN) emerges as the most reported glomerular pathology in end-stage liver disease (ESLD). Additionally, occurrences of membranoproliferative and post-infectious glomerulopathy have been reported. The incidence of secondary IgAN in individuals with cirrhosis remains unknown. The observed prevalence of secondary IgAN may be influenced by variations in renal biopsy practices, especially in ESLD due to the substantial risk of bleeding complications [4]. In a prospective study of 60 patients with ESLD who had trans-jugular kidney biopsies done at the time of liver transplant, morphological abnormalities were identified in 25 of the 48 kidney biopsy samples that were adequate for pathological interpretation. IgAN alone was noted in 25% (12/48), diabetic nephropathy was noted in 21% (10/48), and three had a combination of IgAN and diabetic nephropathy [5].

Another notable glomerular pathology reported (albeit rarely) involves the deposition of lipid particles within the glomeruli, exhibiting similarities to changes observed in individuals with lecithin-cholesterol acyltransferase (LCAT) deficiency [6]. However, little is known about the pathogenesis and natural history of this condition, and the impact of liver transplantation remains unexplored. In this context, we present a case with persistent hematuria and proteinuria post liver transplantation with kidney biopsy revealing hepatic glomerulopathy with features of both IgAN and LCAT deficiency.

## Case presentation

**Figure 1:**
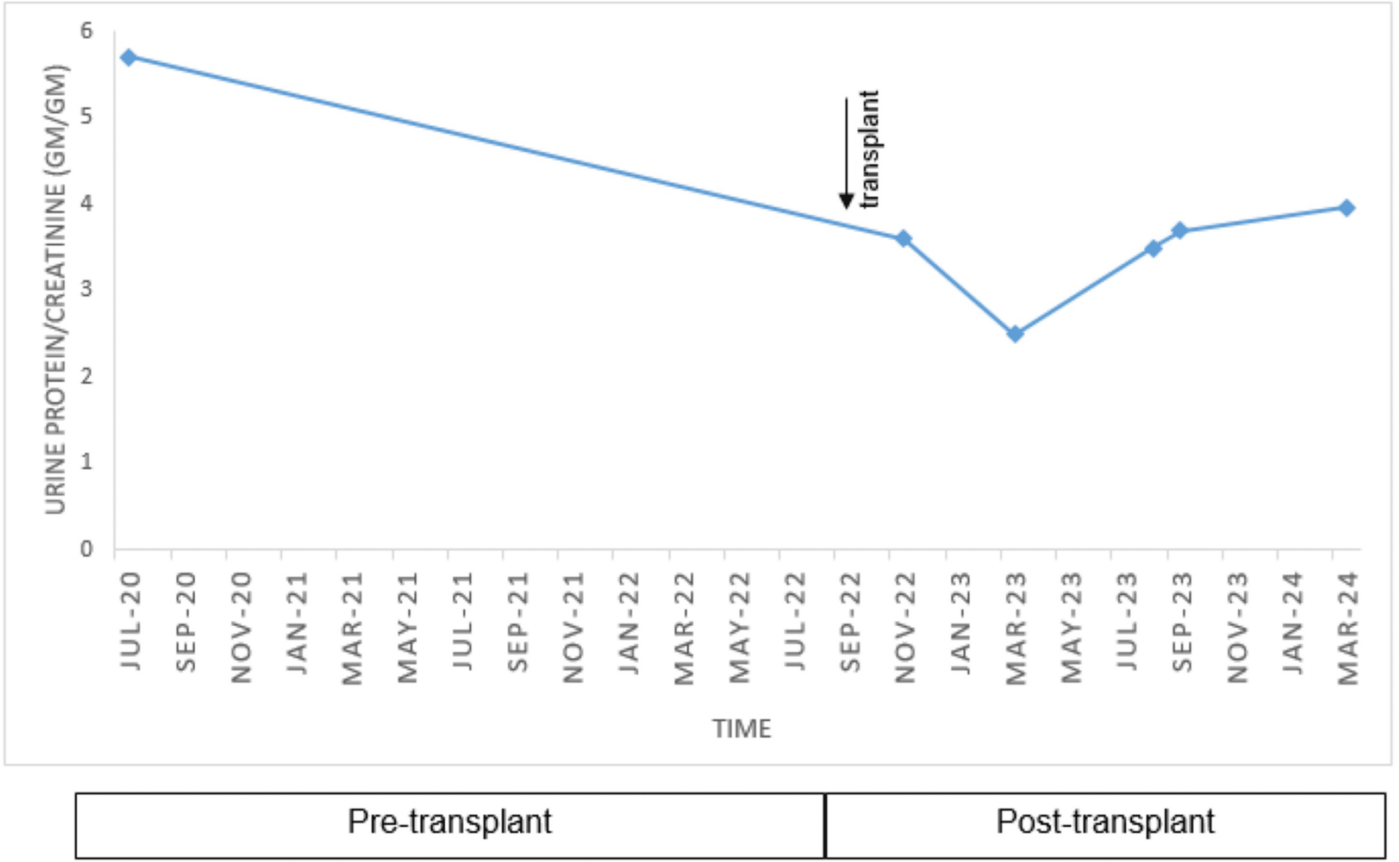
Proteinuria time course

A 58-year-old man with ESLD from alcohol-related liver cirrhosis, complicated by hepatocellular carcinoma, underwent orthotopic liver transplantation (OLT) in September 2022. He was referred to the renal clinic for persistent proteinuria following a liver transplant. He had no known family history of any kidney disease. Prior to the transplant, he experienced multiple episodes of acute kidney injury (AKI), with a serum creatinine level of 1.8 mg/dL on the day of transplantation. Additionally, he was noted to have nephrotic range proteinuria (spot urine protein-creatinine ratio (UPCR) of 5.7 g/g) at least two years prior to the liver transplant. Despite extensive serological investigation, no specific cause was identified. Notably, a kidney biopsy was not performed at that time. His immediate post liver transplant course was complicated by recurrent hydrothorax requiring chest tube placement and biliary anastomotic stricture necessitating stent placement. Despite these challenges, he was discharged one month post transplant in stable condition with mycophenolic acid (360 mg QID) and tacrolimus (trough goal level 6-8 ng/ml) for immunosuppression and a serum creatinine level of 0.7 mg/dL at discharge. Blood pressure (BP) was controlled on nifedipine 60 mg daily and furosemide 40 mg daily, with average BP readings in the 125/75 mm/Hg range post transplant. Physical exam was unremarkable except for +1 pitting pedal edema in bilateral lower extremities. Urine analysis revealed clear urine with pH 6, specific gravity 1.028, +3 protein, negative glucose, 6/HPF RBC, 3/HPF WBC, and three hyaline casts. The repeat spot UPCR was 3.6 g/g. Further workup is shown in Table 1, and the proteinuria time course is shown in Figure 1.

**Table 1:** Workup Na: sodium; K: potassium; LDL: low-density lipoprotein; HDL: high-density lipoprotein; neg: negative; Anti-PLA2R: anti-phospholipase A2 receptor; HIV: human immunodeficiency virus; HBV: hepatitis B virus; HCV: hepatitis C virus; SPEP: serum protein electrophoresis

Lab	Patient values	Reference range	Lab	Patient values	Reference range
Na	139	136-144 mmol/l	Albumin	3.7	3.6-5.0 g/dl
K	4.4	3.3-5.1 mmol/l	Cholesterol	233	<200 mg/dl
Urea nitrogen	18	7-22 mg/dl	LDL	152	<100 mg/dl
Creatinine	0.98	0.6-1.4 mg/dl	HDL	43	>39 mg/dl
Calcium	9.4	8.9-10.3 mg/dl	Triglyceride	236	<150 mg/dl
C3	88.3	90-180 mg/dl	C4	9.2	10-40 mg/dl
Serology					
ANA 1:320	DsDNA neg	SSA/SSB neg	ANCA neg	Anti-PLA2R neg	HIV, HBV, HCV neg
SPEP negative for monoclonal gammopathy					

**Figure 2:**
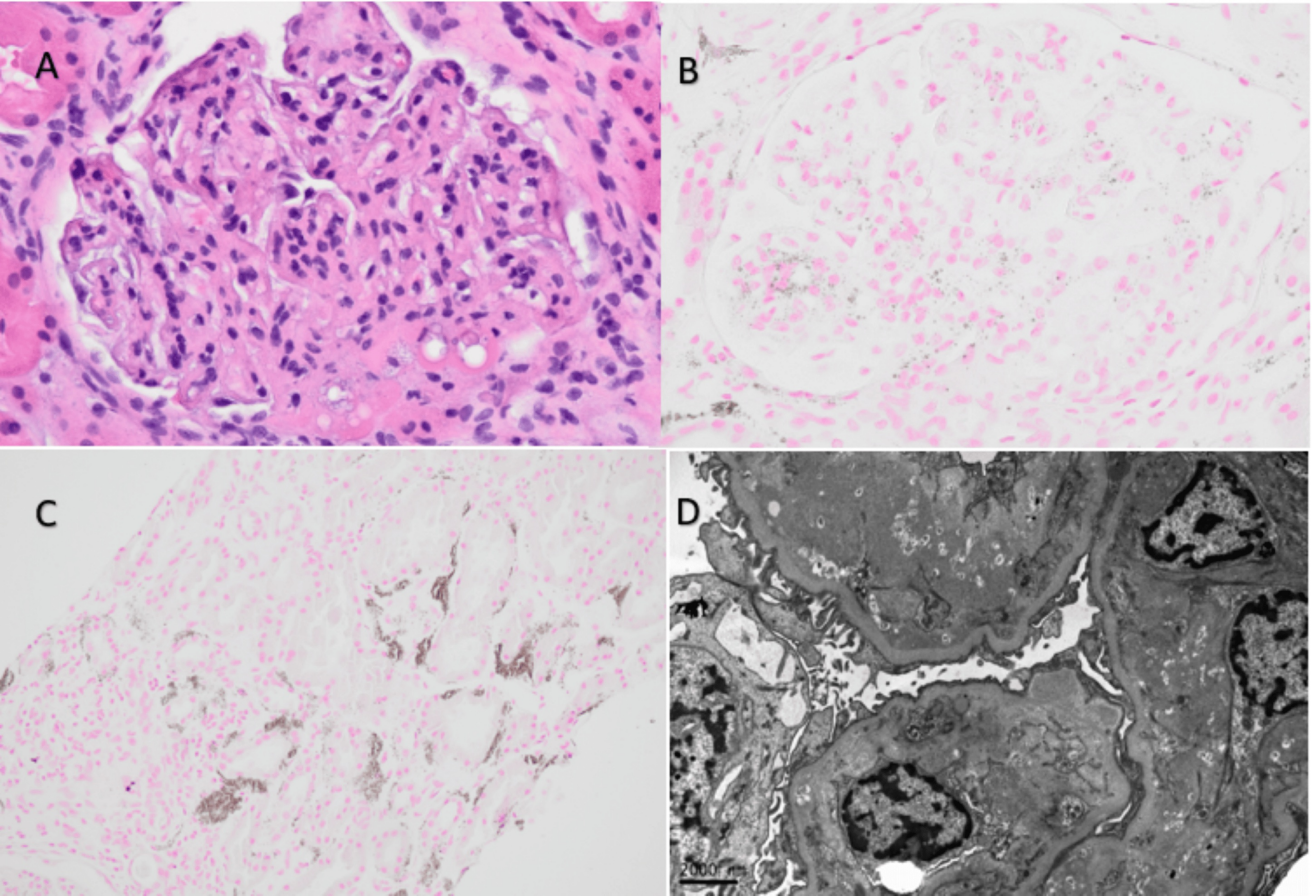
(A) H&E 400×: glomerulus with calcifications and foamy macrophage in the mesangial area. (B) von Kossa calcium stain 400×: glomerulus with calcifications. (C) von Kossa calcium stain 200×: peritubular calcium deposits. (D) EM with intramembranous, subendothelial, and mesangial partly osmiophilic partly lucent deposits consistent with lipid material H&E: hematoxylin and eosin; EM: electron microscopy

Given persistent proteinuria in the context of immunosuppression post liver transplant, kidney biopsy was pursued. Biopsy findings revealed global scleroses in 19% of glomeruli (5/26) on light microscopy, with segmental mesangial increase and thinking of glomerular capillary walls and segmental double contouring in all remaining glomeruli. In addition, the presence of frequent interstitial peritubular and glomerular mesangial microcalcification accompanied by macrophage infiltration was seen with von Kossa and CD68 stains. Immunofluorescence (IF) showed +1/+2 IgA deposition and diffusely positive C4d in the glomeruli along with trace positive staining for IgM. All remaining stains (IgG, kappa and lambda light chain, C3, C1q, fibrinogen, and albumin) were negative or showed a non-diagnostic pattern. EM revealed intramembranous, subendothelial, and mesangial partly osmiophilic/partly lucent deposits consistent with lipid material (Figure 2D). In addition, there were rare mesangial electron-dense deposits that go along with IgA-positive staining on IF and partial effacement of the epithelial cell foot processes (Figure 2).

**Figure 3:**
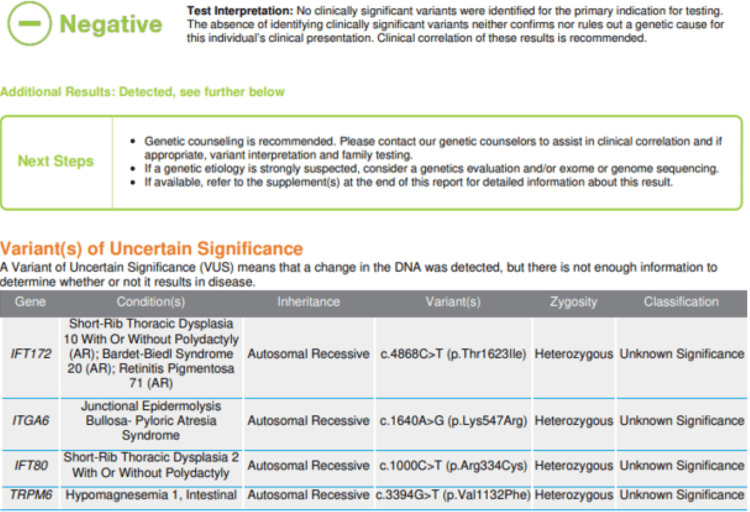
Genetic testing

Genetic testing was also performed but did not reveal any significant known pathogenic variants (Figure 3).

## Discussion

LCAT, an enzyme comprising 416 amino acids, is found in plasma, either bound to lipoproteins or in a lipid-free form. Synthesized primarily in the liver, LCAT plays a crucial role in plasma lipid metabolism by converting lecithin (phosphatidylcholine) and cholesterol into cholesteryl esters. This process helps maintain the balance of unesterified cholesterol between peripheral cells and high-density lipoprotein (HDL) [7]. The liver is central to maintaining cellular and plasma lipid homeostasis. Notably, in liver cirrhosis, there is a significant decrease in LCAT enzyme activity. This reduction is considered a key factor in the pathogenesis of most observed lipoprotein alterations in chronic liver disease [8]. LCAT deficiency is associated with an abnormality in the composition, shape, and concentration of all plasma lipoproteins. This includes low or absent levels of HDL in the plasma, elevated serum free cholesterol, hypertriglyceridemia, the presence of lipoprotein X, reduced levels of apolipoprotein A-II and A-II, and increased levels of apolipoprotein E [9].

Renal involvement in LCAT deficiency commonly manifests as proteinuria, often serving as the initial manifestation. In the initial stages, renal histopathological examination under light microscopy may resemble membranous nephropathy, characterized by mild mesangial expansion and vacuolation of glomerular capillary walls. Silver-stained sections may reveal spikes on the glomerular basement membrane (GBM). As the disease progresses, thickening and vacuolation of the GBM, often with double contouring, become more prominent due to ongoing lipid deposition. Vacuolation may also extend to the Bowman capsule, mesangial matrix, arterioles, and interlobular arteries. IF typically yields negative results, although some patients may exhibit C3 deposition. EM reveals small, irregular, partly osmiophilic, partly lucent lipid deposits in various renal regions, particularly in the subepithelial and subendothelial aspects of the GBM, mesangial matrix, Bowman capsule, and vascular endothelium. Rarely, intraluminal thrombus-like deposits with concentric lamellated substructures may be observed in dilated glomerular capillary loops [6,9]. The light and EM findings in our patient resemble those seen in individuals with LCAT deficiency, although the patient did not have any corresponding biochemical data correlating with LCAT deficiency (absent low-density lipoprotein (LDL) or elevated serum free cholesterol or triglycerides). We suspect the microcalcifications noted on light microscopy are related to prior episodes of AKI as no correlating biochemical abnormalities were noted on blood work.

The histopathological findings of kidney biopsies in LCAT deficiency patients share similarities with those seen in hepatic glomerulosclerosis. Sakaguchi et al. conducted an EM examination of kidney biopsies from 24 patients with liver disease, revealing glomerular changes characterized by the deposition of "black particles," thickening of the GBM, and an increase in the mesangial matrix. Hovig et al. noted that these "black particles" described by Sakaguchi et al. resemble the deposits seen in patients with familial LCAT deficiency and are lipid deposits, suggesting that lipid deposition may play a role in the development of glomerulosclerosis in individuals with liver disease [10,11].

There is a scarcity of data on the clinical course of hepatic glomerulosclerosis post liver transplantation with some reports describing the persistence of IgAN post liver transplantation [12]. There are no published reports on kidney biopsy findings resembling LCAT deficiency post liver transplantation. To our knowledge, our case is the first to be reported. It is hypothesized that in the context of liver transplantation, such renal alterations would resolve. However, there is a lack of data to support or contradict such a hypothesis. It remains uncertain on how long after liver transplantation the lipid deposits in the kidney resolve if at all and what outcomes to expect. There are no specific treatments for hepatic glomerulosclerosis [13]. In our patient, nephrotic range proteinuria and hematuria remain persistent 1.5 years post liver transplant with an otherwise uncomplicated post transplant course.

## Conclusions

Hepatic glomerulosclerosis with kidney biopsy findings resembling LCAT deficiency is rarely reported as kidney biopsy is not often done in ESLD. The clinical course and outcomes post liver transplantation remain unknown. Our case signifies the lack of data and the need for more attention to glomerular pathology post liver transplantation in an era where liver transplantation rates are steadily increasing.
